# A Biomimetic NAC-Loaded PCL/Modified Chitosan/dECM Fibrous Scaffold for Accelerating Diabetic Wound Healing and Minimizing Scarring

**DOI:** 10.3390/polym18040525

**Published:** 2026-02-20

**Authors:** Yiju Xie, Banchao Ruan, Yihua Yin, Lihong Fan, Haolin Tang, Heshuang Dai, Sasha You, Shiyuan Yao, Guangxu Wang, Yihan Xu

**Affiliations:** 1 Sanya Science and Education Innovation Park of Wuhan University of Technology, Sanya 572024, China; 348315@whut.edu.cn (Y.X.); yihuayin@aliyun.com (Y.Y.); thln@whut.edu.cn (H.T.); 358846@whut.edu.cn (S.Y.); 348337@whut.edu.cn (S.Y.); 348334@whut.edu.cn (G.W.); 348336@whut.edu.cn (Y.X.); 2 School of Materials Science and Engineering, Wuhan University of Technology, Wuhan 430070, China; 3 Department of Chemistry, University of Liverpool, Liverpool L69 3BX, UK; sgbruan@liverpool.ac.uk; 4 School of Chemistry, Chemical Engineering and Life Sciences, Wuhan University of Technology, Wuhan 430070, China

**Keywords:** nanofibrous scaffold, diabetic wound, electrospinning, scarless wound healing

## Abstract

The development of innovative wound dressings capable of accelerating diabetic wound healing while simultaneously reducing scar formation is a significant clinical challenge. In this study, we designed and fabricated a multifunctional nanofibrous scaffold PCL/Az-CS/dECM/NAC by incorporating decellularized extracellular matrix (dECM) and N-acetylcysteine (NAC) into a composite backbone of polycaprolactone (PCL) and azidobenzoic acid-modified chitosan (AZCS). The scaffold exhibited ideal hydrophilicity and swelling capacity, and demonstrated excellent biocompatibility. In vitro studies demonstrated that the scaffold effectively scavenged reactive oxygen species (ROS) and promoted the polarization of macrophages from the M1 phenotype to the M2 phenotype; in vivo studies confirmed that the PCL/AZ-CS/dECM/NAC scaffold significantly accelerated wound closure, promoted mature angiogenesis, and facilitated orderly collagen deposition. The PCL/AZ-CS/dECM/NAC scaffold mitigated scar formation by increasing the proportion of regenerative type III collagen, optimizing the collagen I/III ratio. Our findings suggest that the PCL/AZ-CS/dECM/NAC scaffold is a highly promising candidate for a multifunctional dressing designed to treat recalcitrant diabetic wounds and prevent excessive scarring.

## 1. Introduction

Pathological scar formation following skin injury, particularly hypertrophic scars and keloids, represents a formidable clinical challenge, imposing significant physiological and psychological burdens on millions of patients annually [1,2]. Beyond aesthetic disfigurement, the associated contracture, persistent pruritus, and chronic pain severely compromise patients’ quality of life [3,4]. However, despite the availability of various therapeutic modalities, including laser therapy, pressure therapy, and surgical excision, their high costs and persistently high recurrence rates underscore an urgent clinical need [5,6]. This challenge is further exacerbated in chronic conditions such as diabetes, where impaired wound healing itself significantly enhances the risk and severity of scar development [7,8]. Therefore, there is a critical demand for innovative therapeutic strategies capable of simultaneously accelerating refractory wound repair and robustly inhibiting scar formation.

Ideal wound healing progresses through four well-ordered phases: hemostasis, inflammation, proliferation, and remodeling [9,10]. In the early stages of diabetic wound healing, the inflammatory phase is often prolonged and dysregulated due to factors such as infection and hyperglycemia, which leads to a persistent burst of reactive oxygen species (ROS) [11,12]. A highly oxidative microenvironment not only impairs the macrophages’ polarization and the formation of granulation tissue, slowing the healing process [13,14], but also drives the excessive differentiation of fibroblasts into myofibroblasts, which are the key effector cells responsible for secreting and remodeling a disordered extracellular matrix, which leads to scar formation [15,16]. Therefore, a balanced redox state is likely critical for prompt healing [17]. To address this challenge, researchers have explored a variety of antioxidant dressings. For instance, hydrogels loaded with natural antioxidants (e.g., curcumin, resveratrol, berberine) are capable of providing a moist environment and releasing their therapeutic cargo [18,19,20]. However, their efficacy is often limited by the generally poor storage stability of hydrogels and the low solubility of hydrophobic drugs [21,22,23]. Another cutting-edge strategy uses nanozymes (e.g., cerium oxide, zirconium oxide, copper, and silver nanoparticles) in dressings for the sustained catalytic scavenging of ROS [24,25,26,27]. However, concerns regarding their potential long-term biosafety still require further consideration [28,29].

In order to overcome these existing limitations and address the dual challenge of chronic wound healing and scar prevention, electrospun nanofibers emerge as a highly promising platform. An ideal wound dressing must effectively modulate the early inflammatory microenvironment, particularly by scavenging excess ROS [30] as well as providing a conducive moist environment and excellent gas exchange throughout the healing process [31,32]. Electrospinning is a versatile and powerful technique for fabricating porous and submicron-scale fibrous membranes. These biomimetic scaffolds not only closely resemble the structural and morphological cues of the natural extracellular matrix (ECM), but also offer clinical advantages such as excellent biocompatibility, superior air permeability, and efficient exudate absorption [33,34,35]. Moreover, the precisely controlled nanoscale fibrous architecture of electrospun membranes can physically guide cell adhesion, proliferation, and directional migration, thus promoting ordered collagen deposition and tissue remodeling, which is a key mechanism for reducing disorganized scar matrix formation [36,37]. Furthermore, electrospun nanofibers are excellent drug delivery platforms, enabling the sustained release of active ingredients during the critical early healing stages to overcome healing stagnation and prevent pathological scarring [38,39].

To solve these challenges in chronic wound healing and scar prevention, we developed a multifunctional platform with biological activity, good mechanical support, and strong antioxidant properties, which was achieved by strategically integrating multiple biomaterials: porcine decellularized extracellular matrix (dECM) and azidobenzoic acid-modified chitosan (AZ-CS) were co-blended with polycaprolactone (PCL). PCL was selected for its excellent mechanical properties and tunable degradability [40]. Chitosan is key because it helps reduce scarring. It inhibits fibroblast overgrowth by regulating the TGF-β1 and bFGF signaling pathway, thereby preventing excessive myofibroblast differentiation. Due to its structural resemblance to glycosaminoglycans (GAGs), chitosan also regulates the deposition and organization of collagen into a regenerative pattern. Its abundant free amino and hydroxyl groups provide crucial anti-inflammatory and antioxidant effects by neutralizing reactive oxygen species (ROS), which steer the healing process towards regeneration over fibrosis [41,42]. In this study, we utilized azidobenzoic acid-modified chitosan, which retains these inherent biological properties. The partial modification ensures that a significant portion of the active functional groups remain available, while simultaneously introducing light-induced crosslinking stability to the fibrous matrix. This design aimed to provide superior structural stability and a biomimetic substrate. Inherent biological functions that are provided by dECM components promote cellular adhesion, migration, and proliferation [43]. The dECM content was key to the scaffold’s properties; insufficient dECM might compromise the provision of an optimal microenvironment for effective cell growth, while an excessive amount could severely impair the scaffold’s mechanical integrity, spinning uniformity, and overall structural stability. Consequently, a primary scientific objective of this study was to systematically investigate the impact of dECM content on the physicochemical and biological performance of the PCL/AZ-CS/dECM composite scaffold. We aimed to find the best balance between physical strength and biological activity. Building upon the optimized base scaffold, we incorporated N-acetylcysteine (NAC), a strong antioxidant with established pro-angiogenic capabilities [44], to directly address the central challenge of oxidative stress. This approach aimed to establish a powerful synergistic antioxidant system, combining the intrinsic properties of the AZCS biopolymer with the potent scavenging activity of NAC. We hypothesized that this synergistic action would drive the M1-to-M2 macrophage transition and enhance angiogenesis, ultimately accelerating wound closure while minimizing scar formation through optimized collagen remodeling.

In this study, a novel NAC-loaded nanofibrous scaffold was fabricated via electrospinning, using PCL, dECM, and AZ-CS. We characterized the morphology, microstructure, and physicochemical properties of this composite scaffold. In vitro investigations evaluated its biocompatibility, antioxidant activity, anti-inflammatory effects, and capacity to promote crucial cellular behaviors. Subsequently, in vivo studies were meticulously conducted to assess the scaffold’s therapeutic efficacy in accelerating diabetic wound healing in rats and its potent inhibitory effect on hypertrophic scar formation, providing a thorough preclinical evaluation of its potential for transformative clinical translation.

## 2. Materials and Methods

### 2.1. Materials

Fresh porcine tendon was purchased from Wuchang Slaughterhouse (Wuhan, China). Including Triton X100 (97%), sodium dodecyl sulfate (SDS, 99.5% biotech grade) Acetic acid, formic acid, Hexafluoroisopropanol (HFIP), dimethylformamide (DMF), 4-azidobenzoic acid (assay > 97.0%), Etramethylethylenediamine (TEMED, >98.0%), Chitosan (CS, MW = 100,000), 1-(3-Dimethylaminopropyl)-3-ethylcarbodiimide (EDC, ≥98.0%), PCL, N-acetylcysteine (NAC), phosphate-buffered saline (PBS), N, N-Dimethylformamide (DMF, ≥99%), Methylene blue, Nitrotetrazolium Blue (NBT), Phenazine Methyl Sulfate (PMS), Tris, Reduced Coenzyme I (NADH), 30% hydrogen peroxide (H_2_O_2_), and FeSO_4_ (≥98%), all reagents used in this study were obtained from Shanghai Macklin Biochemical Co., Ltd. (Shanghai, China).

### 2.2. Preparation of Decellularized Extracellular Matrix

The decellularized extracellular matrix (dECM) was prepared according to the previous modified methods [45]. The fresh porcine tendon tissues were cut into 0.5 cm × 0.5 cm slices and washed using PBS for 2 h. The sliced tissues were soaked in a 0.5% SDS and 0.5% Triton X-100 and stirred for 8 h. After that, the soaked tissues were washed thoroughly using PBS again. Next, the tissues were treated with 1% HAc solution for 24 h and washed using distilled water for 48 h. Lastly, the tissues were freeze-dried.

### 2.3. Synthesis of Azidobenzoic Acid-Modified Chitosan

Azidobenzoic acid was conjugated to the chitosan backbone to engineer a photo-crosslinkable derivative (AZCS). This modification was specifically designed to enhance the structural integrity of the electrospun nanofibers while maintaining the intrinsic biological functions of chitosan. According to the previous methods [46]. First, 4 g of chitosan was dispersed in 300 mL of distilled water, followed by the addition of a 10 mL aqueous solution containing 1.162 g of TEMED. Subsequently, 1.4 g of EDC was dissolved in 20 mL of distilled water and 0.8 g of 4-azidobenzoic acid was dissolved in 5 mL of DMSO. This mixture was then added to the chitosan suspension. After adjusting the pH to 5 with HCl, the reaction was stirred overnight at room temperature. The resulting solution was then purified using a dialysis membrane (10 kDa MWCO, Labshark, Changde, China) against distilled water for 3 days. Finally, the solution was lyophilized overnight to obtain the dry Az-CS powder.

### 2.4. Characterization of the Decellularized Matrix

Histological experiments (H&E staining) were performed on samples prepared from native porcine Achilles tendon and decellularized porcine Achilles tendon to evaluate the efficacy of the decellularization process. The experiments were conducted following standard procedures for fixation, H&E staining, and microscopic observation.

### 2.5. Characterization of Az-CS

Fourier Transform Infrared (FT-IR) spectroscopy was used for analysis. The FTIR spectra were obtained within the range between 500 and 4000 cm^−1^ with a resolution of 1 cm^−1^, with 64 scans for each spectrum.

### 2.6. Preparation of Electrospun Fibrous Scaffolds

**Figure 1:**
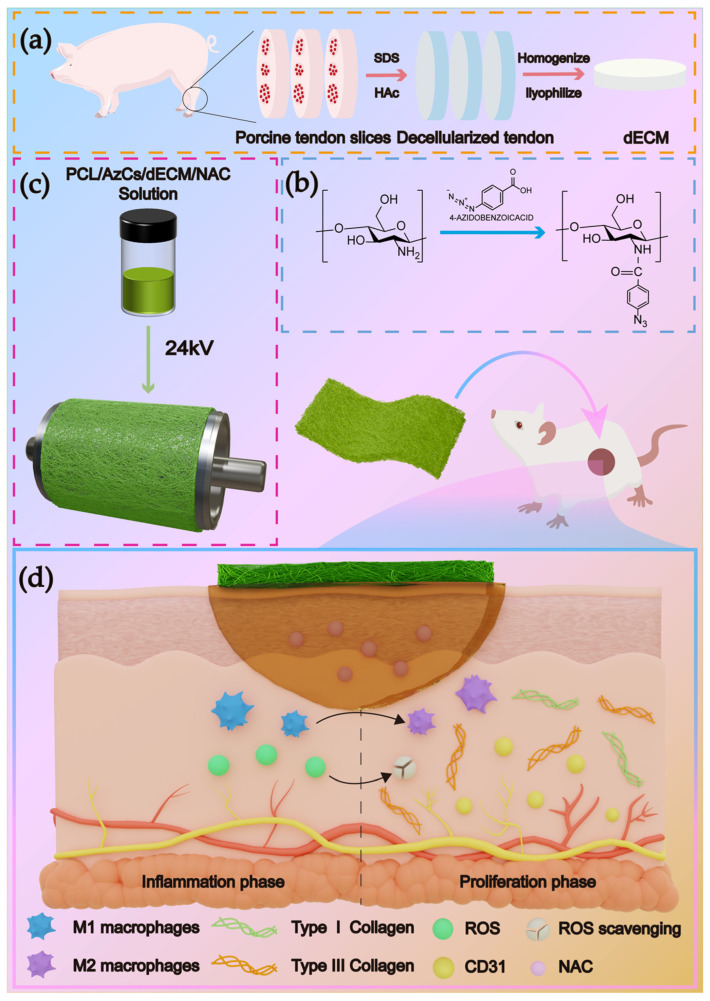
(**a**) Preparation of porcine tendon-derived dECM. (**b**) Synthesis of AZ-CS. (**c**) Fabrication of the PCL/AzCS/dECM/NAC composite scaffold via electrospinning. (**d**) Proposed mechanism of PCL/AZ-CS/dECM/NAC-mediated diabetic wound healing. The released NAC scavenges excess ROS and drives M1-to-M2 macrophage polarization, accelerating the transition to the proliferative phase. This process enhances angiogenesis (CD31) and balances Collagen I/III deposition, leading to rapid wound closure and reduced scarring.

A mixture of 1 g of Az-CS and PCL (*w*/*w* = 1:9) along with dECM (at 5, 7, or 9 *w*/*w*%) was added to 10 mL of a mixed solvent consisting of formic acid, hexafluoroisopropanol (HFIP), and dimethylformamide (DMF) (1:8:1, *v*/*v*/*v*). The choice of solvents was tailored to ensure the complete dissolution of all components: formic acid was utilized to dissolve the modified chitosan (Az-CS), DMF was incorporated to ensure the solubility of the NAC drug, and HFIP served as the primary solvent for both the PCL matrix and the dECM. The mixture was dissolved by stirring at 1000 rpm at 40 °C. After complete dissolution, 0.1 g of N-acetylcysteine was added, and the solution was stirred until homogeneous. The final solution (10 mL) was drawn into a syringe fitted with a 21-gauge needle. Electrospinning was performed at 37 °C and 40% relative humidity under the following optimized conditions: an applied voltage of 24 kV, a constant flow rate of 0.8 mL/h, and a tip-to-collector distance of 15 cm. The nanofibers were deposited onto a rotating drum collector (15 cm in diameter and 35 cm in length) operating at a speed of 800 rpm, with the effective collection width set to 15 cm. After spinning, the collected fibrous scaffolds were dried overnight in a vacuum oven at 37 °C (Figure 1).

### 2.7. Drug Release Profile Testing

The drug loading content and cumulative NAC release from the scaffolds were quantified using UV-Vis spectrophotometry at a wavelength of 211 nm. For drug loading content, precisely weighed drug-loaded nanofiber membranes (in triplicate) were extracted in water for 4 h using an ultrasonicator. The extract was then centrifuged at 10,000 rpm for 10 min. The absorbance of the supernatant was measured at 211 nm using a UV/Vis spectrophotometer. For cumulative drug release analysis, scaffold samples (20 mg) were immersed in 10 mL of PBS. At specified time points (0, 1, 2, 3, 6, 8, 12, 24, 36, 48, 72, 96, and 120 h), the concentration of NAC in the release medium was calculated based on the absorbance at 211 nm.

### 2.8. In Vitro Antioxidant Activity Assays

#### 2.8.1. Superoxide Radical Scavenging Assay

The superoxide radical (O_2_^−^•) scavenging activity was determined using the PMS-NADH method as described by Yang [47], with minor modifications. First, to ensure complete drug release, 0.1 g of the sample was suspended in 3.0 mL of phosphate-buffered saline (PBS) and sonicated at 37 °C for 4 h. For the assay, the sample solution (3.0 mL) was mixed with 1.0 mL of nicotinamide adenine dinucleotide (NADH) solution, 1.0 mL of nitro blue tetrazolium (NBT) solution, and 1.0 mL of phenazine methosulfate (PMS) solution. The total volume of the reaction system was 6.0 mL. The control group was prepared using 3.0 mL of PBS instead of the sample solution, mixed with 1.0 mL of NADH, 1.0 mL of NBT, and 1.0 mL of PMS. The blank group contained 4.0 mL of PBS, 1.0 mL of NBT, and 1.0 mL of PMS (without NADH).

All mixtures were incubated in a water bath at 37 °C for 30 min. The absorbance of each solution was subsequently measured at 259 nm using a UV-Vis spectrophotometer. The scavenging activity was evaluated based on the inhibition of NBT reduction. All experiments were performed in triplicate. The scavenging efficiency was calculated using the following formula:

(1) $$Scavenging\;Activity\;\left(\%\right)=\;\left[\frac{A_{sample\;259\;nm}-A_{control\;259\;nm}}{A_{blank\;259\;nm}}\right]\times 100\%$$

#### 2.8.2. Hydroxyl Radical Scavenging Assay

The hydroxyl radical (•OH) scavenging activity was evaluated by monitoring the degradation of methylene blue via a Fenton-type reaction. A 0.1 g sample was first sonicated in 3.0 mL of PBS at 37 °C for 4 h to release the drug. The resulting sample solution (3.0 mL) was then mixed with 1.0 mL of EDTA-Fe (II) solution, 2.0 mL of methylene blue solution, and 2.0 mL of 3% H_2_O_2_ solution, reaching a final volume of 8.0 mL. The blank group, representing maximal absorbance, contained 3.0 mL of PBS, 1.0 mL of EDTA-Fe (II), 2.0 mL of methylene blue, and 2.0 mL of deionized water (in place of H_2_O_2_). The control group, representing maximal radical-induced degradation, contained 3.0 mL of PBS, 1.0 mL of EDTA-Fe (II), 2.0 mL of methylene blue, and 2.0 mL of 3% H_2_O_2_. The mixtures were incubated at 37 °C for 30 min, after which the absorbance was measured at 663 nm. All assays were conducted in triplicate. The hydroxyl radical scavenging efficiency was calculated using the formula below:

(2) $$Scavenging\;Activity\;(\%)\;=\;[\frac{A_{sample\;663\;nm}-A_{control\;663\;nm}}{A_{blank\;663\;nm}}]\times 100\%$$

### 2.9. In Vivo Animal Experiments

#### 2.9.1. Evaluation of In Vivo Wound Healing Performance

To induce diabetes, male Sprague-Dawley (SD) rats (6 weeks old, weighing 150–190 g) received a single intraperitoneal injection of streptozotocin (STZ, 60 mg/kg) in 0.1 M citrate buffer (pH 4.5) after a 12 h fast. Five days after induction, blood glucose levels were monitored using a tail-vein blood sample. Only animals with fasting blood glucose levels exceeding 16.5 mmol/L, indicating that the type I diabetes model was successfully established, were classified as diabetic and included in the subsequent wound healing study. All surgical procedures were conducted under general anesthesia. SD diabetic rats were anesthetized with 2% sodium pentobarbital. Following anesthesia, the rats were immobilized in a prone position, and the hair on the dorsal mid-spinal region was completely removed using a depilatory cream. Two standardized circular, full-thickness skin wounds (15 mm in diameter) were then created on the side of the spine. The rats were randomly assigned to five experimental groups (*n* = 3 per group): control (treated with sterile saline), PCL, PCL/AZ-CS, PCL/AZ-CS/dECM, and PCL/AZ-CS/dECM/NAC. The wound healing process was monitored by capturing digital photographs at designated time points (days 0, 3, 7, 10, and 14 post-treatment). The wound contraction rate was quantitatively analyzed using ImageJ software (version 1.49) and calculated according to the following formula:

(3) $$Wound\;contraction\;rate\;\left(\%\right)=\frac{S_{0}\;-\;S_{t}}{S_{0}}\;\times \;100\%$$

where *S*_0_ is used to represent the original wound area and *S_t_* is used to represent the wound area on day *t*.

All animal experiments were approved by the Animal Ethics Committee of Wuhan Myhalic Biotechnology Co., Ltd., Wuhan, China (Approval No. HLK-20250701-001) and strictly complied with the “Guidelines for the Use and Management of Laboratory Animals” issued by the National Institutes of Health (NIH) of the USA and the “Implementation Rules for the Management of Medical Laboratory Animals” issued by the National Health and Family Planning Commission of China.

#### 2.9.2. Histological and Immunohistochemical Analysis

At day 14 post-treatment, the rats were euthanized, and the wound tissues, along with the surrounding healthy skin, were harvested. The collected specimens were fixed in 4% paraformaldehyde, dehydrated through a graded ethanol series, and subsequently embedded in paraffin blocks. The samples were then sectioned for Hematoxylin and Eosin (H&E) and Masson’s trichrome staining. To further assess the quality of wound healing and the underlying biological mechanisms, immunohistochemical (IHC) staining was performed for various markers, including CD86 (M1 phenotype), CD206 (M2 phenotype), CD31 (angiogenesis), and α-smooth muscle actin (α-SMA). Additionally, Sirius Red staining was utilized to differentiate and quantify the distribution of Collagen type I and Collagen type III. All stained sections were observed and imaged using an optical microscope.

### 2.10. Other Parts of the Experimental Section

Further experimental details can be found in the Supplementary Materials.

## 3. Results and Discussion

### 3.1. Examination of the Decellularization Effect of ECM

**Figure 2:**
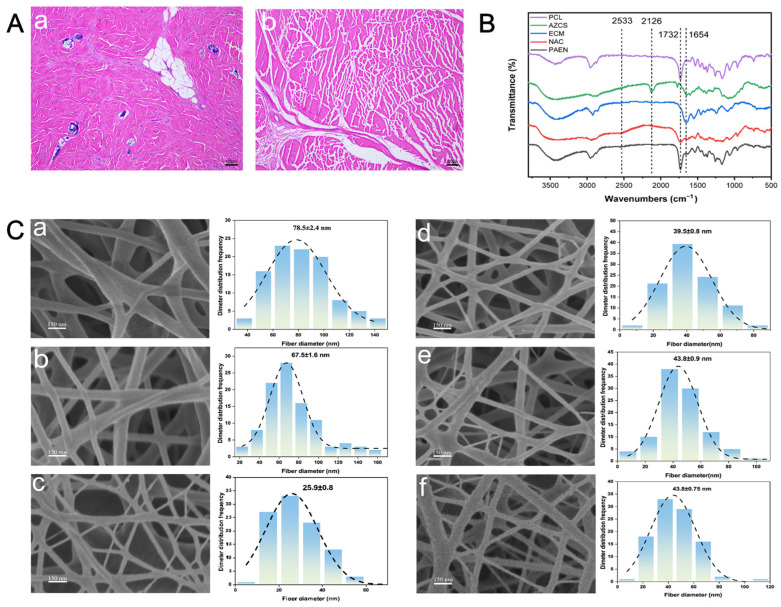
(**A**) HE staining test of decellularization effect. (**a**) HE staining of pig tendon sections. (**b**) HE staining of acellular pig tendon sections. (**B**) FTIR spectra of PCL, AZ-CS, dECM, NAC, and the composite product PCL/AZ-CS/dECM/NAC. (**C**) SEM images and diameter distribution histograms of nanofibers with different compositions. (**a**) PCL, (**b**) PCL/AZ-CS, (**c**) PCL/AZ-CS/3%dECM, (**d**) PCL/AZ-CS/5%dECM, (**e**) PCL/AZ-CS/7%dECM, (**f**) PCL/AZ-CS/7%dECM/NAC. Scale bar: 150 nm.

The introduction of allogeneic cells into the body can elicit severe immune rejection and adverse reactions [48]. Therefore, when utilizing ECM from allogeneic sources, ensuring the complete removal of cellular components from the tissue is crucial. To verify the efficacy of decellularization, H&E staining was performed on the porcine tendon before and after the process. The H&E stained image of the native porcine tendon (Figure 2Aa) revealed the presence of cellular nuclei, stained bluish-purple. In contrast, the H&E image of the decellularized porcine Achilles tendon (Figure 2Ab) appeared pink, with a near-complete absence of cells. These H&E staining images indicate the successful preparation of a decellularized porcine tendon.

It should be noted that while the dECM preparation protocol used in this study has been validated in our previous work [45] to meet standard decellularization criteria, we did not perform batch-specific DNA quantification or biochemical analysis for the current study. This represents a limitation, particularly regarding potential batch-to-batch variability and immunogenicity, which will be more rigorously addressed in our future investigations.

### 3.2. Properties of the PCL/AZ-CS/dECM/NAC Nanofiber Scaffold

#### 3.2.1. ATR-FTIR Analysis

Individual components were first analyzed via FTIR (Figure 2B). PCL (purple line) exhibited its signature ester carbonyl (C=O) stretching peak at 1732 cm^−1^. For AZ-CS (green line), the sharp peak at 2126 cm^−1^ (asymmetric -N_3_ stretching) confirmed successful azide modification, alongside the amide I band (C=O stretching) at 1654 cm^−1^. The protein-rich dECM (blue line) displayed characteristic amide I and II bands at 1654 cm^−1^ and 1550 cm^−1^, respectively. NAC (red line) showed a distinct -SH stretching vibration at 2533 cm^−1^, with a strong absorption at 1654 cm^−1^ originating from its amide I and carboxyl groups. The PCL/AZ-CS/dECM/NAC spectrum integrated the characteristic features of all components. The 1732 cm^−1^ peak confirmed PCL was included, while the small but still visible peak at 2533 cm^−1^ indicated successful NAC loading. Notably, the azide peak at 2126 cm^−1^ significantly diminished, likely due to photo-induced crosslinking during electrospinning. In normal light, azide groups may form reactive nitrene intermediates that undergo C-H or N-H insertion with dECM amino acid residues, which lead to crosslinking between AZ-CS and the protein matrix [49]. The intense broad peak at 1654 cm^−1^ represented the superposition of amide I bands from dECM, AZ-CS, and NAC, confirming their successful integration into the composite.

#### 3.2.2. Fiber Diameter Analysis

The morphology and diameter of the electrospun nanofibers were analyzed via SEM and ImageJ (Figure 2C). All formulations yielded uniform, bead-free nanofibers forming a randomly oriented 3D porous network. This architecture mimics the natural extracellular matrix (ECM) topology, providing essential physical support for cell behavior, while the protonated amino groups of chitosan further enhance cell adhesion through electrostatic interactions [50].

The average diameter of pure PCL fibers (78.5 ± 2.4 nm) significantly decreased to 67.5 ± 1.6 nm with AZ-CS addition (Figure 2Cb). This was likely due to increased solution conductivity and charge density [51]. Higher conductivity enhances jet stretching within the electric field, resulting in finer fibers. The diameter hit its lowest point (25.9 ± 0.8 nm) after adding 3% dECM (Figure 2Cc). As a biological polyelectrolyte rich in charged proteins and glycosaminoglycans, [52,53]. This led to a sharp increase in electrostatic repulsion, causing the jet to be extremely stretched and resulting in the formation of fine nanofibers. A rebound in fiber diameter was observed as dECM concentration increased to 5% (39.5 ± 0.8 nm) and 7% (43.8 ± 0.9 nm). This happened because of the balance between conductivity and viscosity. Although higher dECM concentrations continue to improve conductivity, they increase macromolecular chain entanglement (collagen, elastin, etc.), which leads to a sharp rise in viscosity [54]. In the 5–7% range, the increased flow resistance from viscosity becomes the dominant factor over electrostatic stretching, ultimately producing thicker fibers.

#### 3.2.3. Mechanical Properties

**Figure 3:**
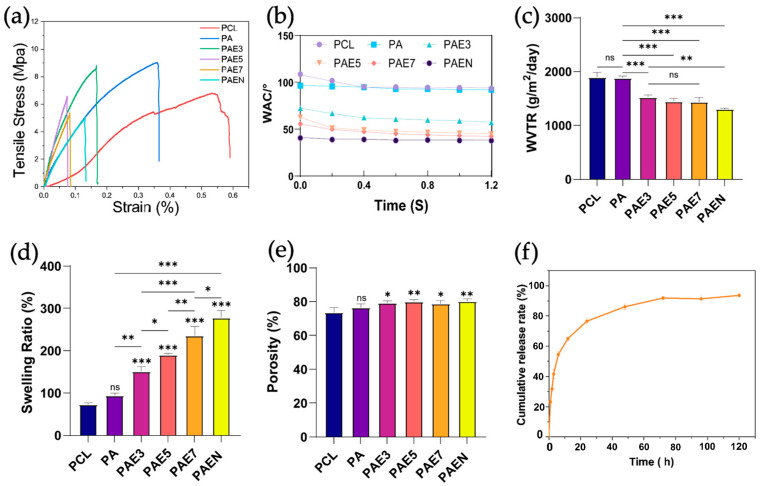
Characterization of the physicochemical properties of the fabricated nanofiber scaffolds. (**a**) Representative stress–strain curves. (**b**) Dynamic water contact angles (WCA). (**c**) Water vapor transmission rate (WVTR). (**d**) Swelling ratio after 24 h immersion in PBS. (**e**) Porosity water vapor transmission rate (WVTR). (**f**) In vitro cumulative release of NAC from the PCL/AZ-CS/dECM/NAC scaffold over 120 h. (Data are presented as mean ± SD (*n* = 3). * *p* < 0.033, ** *p* < 0.002, *** *p* < 0.001. ns, not significant.) Note: PCL: pure polycaprolactone; PA: PCL/Az-CS; PAE3/5/7: PCL/Az-CS with 3, 5, or 7 wt% dECM; PAEN: the final PCL/Az-CS/dECM/NAC multifunctional scaffold.).

The mechanical properties of the composite scaffolds were evaluated via uniaxial tensile testing (Figure 3a). Pure PCL showed a relatively low tensile strength (~6.5 MPa) and elastic modulus (22.2 MPa), but the highest ductility (58% elongation at break) (Figure S1). Adding AZCS significantly enhanced strength and modulus to 9.49 ± 0.34 MPa and 27.99 ± 0.99 MPa, respectively, while elongation dropped to ~37%. A sharp jump in modulus (114.6 MPa) occurred with 3% dECM (PCL/AZ-CS/dECM3), making the material quite stiff but less tough (22% elongation). However, adding more dECM (PCL/AZ-CS/dECM5 and PCL/AZ-CS/dECM7) actually started to weaken both strength and modulus. The final PCL/AZ-CS/dECM/NAC scaffold showed a much better balance, with 5.0 ± 0.18 MPa strength, 44.25 ± 1.58 MPa modulus, and ~16% elongation. These mechanical trends reflect the microstructural evolution of the composites. While PCL provides a flexible matrix, AZCS acts as a reinforcing phase that restricts polymer chain slippage, which increases stiffness but cuts down on stretchability. At 3% dECM functions as an efficient filler; when coupled with the ultra-fine fiber network observed by SEM, it creates a rigid network that spikes the modulus. Conversely, excessive dECM concentrations may lead to macromolecular agglomeration or phase separation, creating stress concentration points that weaken the material. Notably, the PCL/AZ-CS/dECM/NAC scaffold’s modulus is very close to that of natural skin [55]. This blend of moderate strength and skin-like elasticity makes PCL/AZ-CS/dECM/NAC a great fit for wound dressings, as it can handle physical stress while still staying flexible on the skin.

#### 3.2.4. Water Vapor Transmission Rate and Contact Angle

Surface wettability was evaluated via dynamic water contact angle (WCA) measurements (Figure 3b). Pure PCL exhibited inherent hydrophobicity, with a WCA of 93.8 ± 1.3° at 1.2 s, consistent with its lack of hydrophilic groups. The addition of AZCS only slightly reduced the WCA to 92.1 ± 0.7°; a change that is largely within error limits compared to pure PCL. The surface remained hydrophobic, likely due to the dominance of PCL on the fiber surface. A dramatic transition from hydrophobic to hydrophilic was observed upon dECM introduction. The WCA dropped to 57.8 ± 1.2° for the PCL/AZ-CS/dECM3 group and further decreased to 45.5 ± 1.7° (PCL/AZ-CS/dECM5) and 43.0 ± 0.8° (PCL/AZ-CS/dECM7) in a concentration-dependent manner. This enhancement is attributed to the abundant polar moieties (e.g., -COOH, -NH_2_, and -OH) in dECM, which facilitate hydrogen bonding with water. The loading of NAC (PCL/AZ-CS/dECM/NAC group) achieved the highest hydrophilicity (38.0 ± 2.3°) by further increasing the density of polar sites via its carboxyl and amide groups. As shown in the dynamic curves, all samples exhibited a time-dependent WCA reduction, reflecting water penetration and spreading within the porous network—a trend more pronounced in the hydrophilic PCL/AZ-CS/dECM and PCL/AZ-CS/dECM/NAC groups. Overall, the systematic integration of hydrophilic biomolecules into the PCL matrix effectively modulates surface wettability. The superior hydrophilicity of the PCL/AZ-CS/dECM/NAC scaffold (WCA ≈ 38°) is critical for promoting cell adhesion and spreading [56], underscoring its potential as an advanced wound dressing.

The water vapor transmission rate (WVTR) is critical for wound dressings to manage exudate and prevent tissue maceration [57]. As shown in Figure 3c, pure PCL exhibited the highest WVTR (1896.6 ± 86.9 g/m^2^/day), owing to its hydrophobic nature and porous architecture that facilitates vapor diffusion. AZCS incorporation caused only a marginal, non-significant reduction to 1878.4 ± 39.0 g/m^2^/day, suggesting minimal impact on gas permeability. In contrast, dECM addition induced a significant, dose-dependent decrease in WVTR, with values for PCL/AZ-CS/dECM3, PCL/AZ-CS/dECM5, and PCL/AZ-CS/dECM7 dropping to 1523.0 ± 41.3, 1445.5 ± 56.1, and 1438.6 ± 87.5 g/m^2^/day, respectively. This decline is attributed to reduced fiber diameters and smaller pore sizes (consistent with SEM findings) that increase diffusion resistance, and the abundance of hydrophilic moieties (-OH, -NH_2_, -COOH) in dECM and AZCS that bind water molecules, thereby slowing free diffusion. Finally, NAC loading (PCL/AZ-CS/dECM/NAC) further reduced the WVTR to 1301.7 ± 18.1 g/m^2^/day, likely because NAC, as a hydrophilic small molecule, fills interstitial pores or coats fiber surfaces, further obstructing vapor transport.

#### 3.2.5. Swelling Ratio and Porosity

The swelling ratio of the different nanofiber scaffolds was measured after 24 h of immersion in phosphate-buffered saline (PBS), with the results shown in Figure 3d. The pure PCL scaffold exhibited the lowest swelling ratio, at only 73.6 ± 3.2%, which is consistent with its inherent hydrophobicity as demonstrated by the WCA tests. The PCL polymer chains lack functional groups that strongly interact with water molecules; therefore, its water uptake capacity is primarily dependent on the filling of the porous network by water, rather than absorption by the material itself. Upon the addition of AZCS, the swelling ratio slightly increased to 94.3 ± 5.7%, attributed to the introduction of hydrophilic components. Adding dECM significantly boosted the scaffold’s swelling ratio. At 3% dECM, the ratio climbed to 164.4 ± 30.3%. This upward trend followed the dECM concentration, reaching 202.7 ± 10.4% and 259.1 ± 14.7% for the 5% and 7% groups, respectively. These findings align with our WCA data, likely because the polar functional groups in dECM readily form hydrogen bonds with water molecules. The highest swelling ratio (290.9 ± 16.2%) was observed after loading NAC into the PCL/AZ-CS/dECM7 scaffold. The hydrophilic nature of NAC creates even more sites for water interaction, maximizing the scaffold’s ability to soak up liquid.

The porosity of the scaffolds was evaluated to assess their structural suitability (Figure 3e). Pure PCL exhibited a porosity of 73.69 ± 2.93%, which increased non-significantly to 76.35 ± 2.40% upon AZCS introduction. Notably, incorporating dECM led to a stepwise enhancement in porosity, with PCL/AZ-CS/dECM3, PCL/AZ-CS/dECM5, and PCL/AZ-CS/dECM7 reaching 79.21 ± 1.33%, 79.93 ± 1.30%, and 78.71 ± 1.96%, respectively. The NAC-loaded PCL/AZ-CS/dECM/NAC scaffold reached a peak porosity of 80.12 ± 1.51%. This upward trend aligns with the structural changes observed via SEM, where dECM was shown to significantly thin the fibers. The random arrangement of these finer nanofibers creates a 3D network with more empty space and a larger surface area, effectively boosting the overall porosity. A porosity level around 80% is widely considered ideal for tissue engineering [58], as it allows cells to migrate inward and ensures the easy exchange of nutrients and metabolic waste. This well-developed pore structure is a key reason behind the scaffold’s excellent biological performance.

#### 3.2.6. Release Profile of NAC

We studied the NAC release profile from the PCL/AZ-CS/dECM/NAC scaffold to evaluate its potential as a drug carrier (Figure 3f). The release followed a classic two-stage pattern: a rapid initial burst followed by a period of sustained release. Specifically, 65.1% of the NAC was released within the first 12 h, rising to 76.6% after 24 h. The release rate then slowed, hitting a plateau at 72 h and finishing with a cumulative release of 93.6% at 120 h. To understand the release kinetics, the first 60% of the data was fitted to the Korsmeyer–Peppas model [59]. The Peppas equation fits the data well, with a high correlation coefficient (R^2^) of 0.9971.

The release exponent *n* calculated from the model fitting was 0.4729 (±0.027). As this value falls within the range of 0.45 < *n* < 0.89, it indicates that the release of NAC follows a non-Fickian diffusion mechanism [60]. This suggests that the process is driven by both drug diffusion and the swelling or relaxation of the polymer matrix, which is closely linked to the PCL/AZ-CS/dECM/NAC system’s physicochemical properties. As confirmed by the previous swelling ratio and contact angle tests, the PCL/AZ-CS/dECM/NAC scaffold possesses excellent hydrophilicity and a high swelling capacity. When the scaffold comes into contact with PBS buffer, the hydrophilic groups from the AZCS, dECM, and NAC components promote the rapid penetration of water into the fiber network, causing the polymer matrix to swell rapidly. This swelling, on one hand, creates channels for the outward diffusion of NAC molecules, while on the other hand, the relaxation of polymer chains also accelerates drug release.

#### 3.2.7. Antioxidant Activity

**Figure 4:**
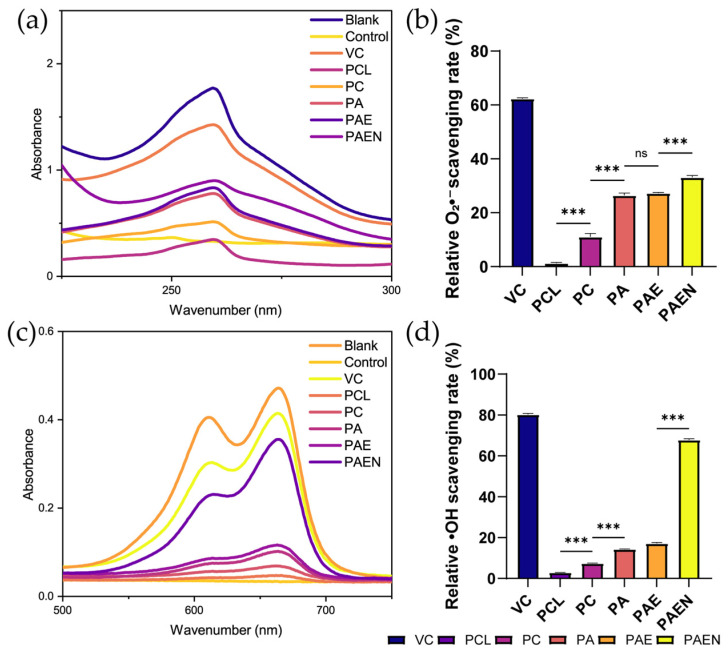
In vitro antioxidant activity of the nanofiber scaffolds. (**a**) UV-Vis absorption spectra for the superoxide anion radical (O_2_•^−^) scavenging assay. (**b**) Corresponding quantitative analysis of the relative O_2_•^−^ scavenging rate. (**c**) UV-Vis absorption spectra for the hydroxyl radical (•OH) scavenging assay. (**d**) Corresponding quantitative analysis of the relative •OH scavenging rate. (Data are presented as mean ± SD (*n* = 3). *** *p* < 0.001. ns, not significant.) Note: PCL: pure polycaprolactone; PA: PCL/Az-CS; PAE: PCL/Az-CS with 7 wt% dECM; PAEN: the final PCL/Az-CS/dECM/NAC multifunctional scaffold.

As a highly reactive oxygen species, the hydroxyl radical (·OH) critically impedes wound healing when produced in excess [61]. This is particularly evident in the chronic wound microenvironment, where M1-polarized phagocytes are dominant. At the wound margins, these M1 cells—which make up about 80% of the total phagocytic population—release high amounts of ·OH [62]. This continuous release keeps the tissue in a state of oxidative damage and blocks the healing process. Therefore, stopping the production of ·OH is vital for lowering oxidative stress and bringing the redox state back to balance. We tested the scaffolds’ ability to scavenge hydroxyl and superoxide anion radicals to check their antioxidant potential. The positive control (VC) cleared 80.22 ± 0.595% of radicals. As seen in Figure 4b,d, pure PCL had almost no effect. In contrast, unmodified chitosan (PC group) showed some ability to scavenge ·OH (7.37 ± 0.15%) and superoxide anions (11.06 ± 1.22%). Notably, adding azide–benzoic acid (PCL/AZ-CS group) enhanced these rates to 14.26 ± 0.22% and 26.46 ± 0.86%, respectively. The NAC-loaded PCL/AZ-CS/dECM/NAC scaffold performed the best, scavenging 67.76 ± 0.58% of ⋅OH and 33.09 ± 0.75% of superoxide anions. These data clearly elucidate the origin of the antioxidant properties of the composite scaffolds.

The comparison between the PC and PCL/AZ-CS groups is mechanistically significant. While chitosan inherently scavenges radicals via its amino and hydroxyl groups [63], azide–benzoic acid modification significantly amplifies this intrinsic capacity, consistent with previous findings [47]: at all tested concentrations, benzoic acid-derivative-modified chitosan exhibited higher hydroxyl radical scavenging activity than CS. Furthermore, the exceptional antioxidant activity exhibited by the final PCL/AZ-CS/dECM/NAC product is the result of the combined effect of AZCS and the potent antioxidant NAC. Through its sulfhydryl (-SH) side chain, NAC interacts with the electrophilic moieties of reactive oxygen species, facilitating the direct neutralization of hydroxyl radicals (·OH) and hydrogen peroxide (H_2_O_2_) [64,65].

### 3.3. Biocompatibility Text of Nanofiber Scaffolds

**Figure 5:**
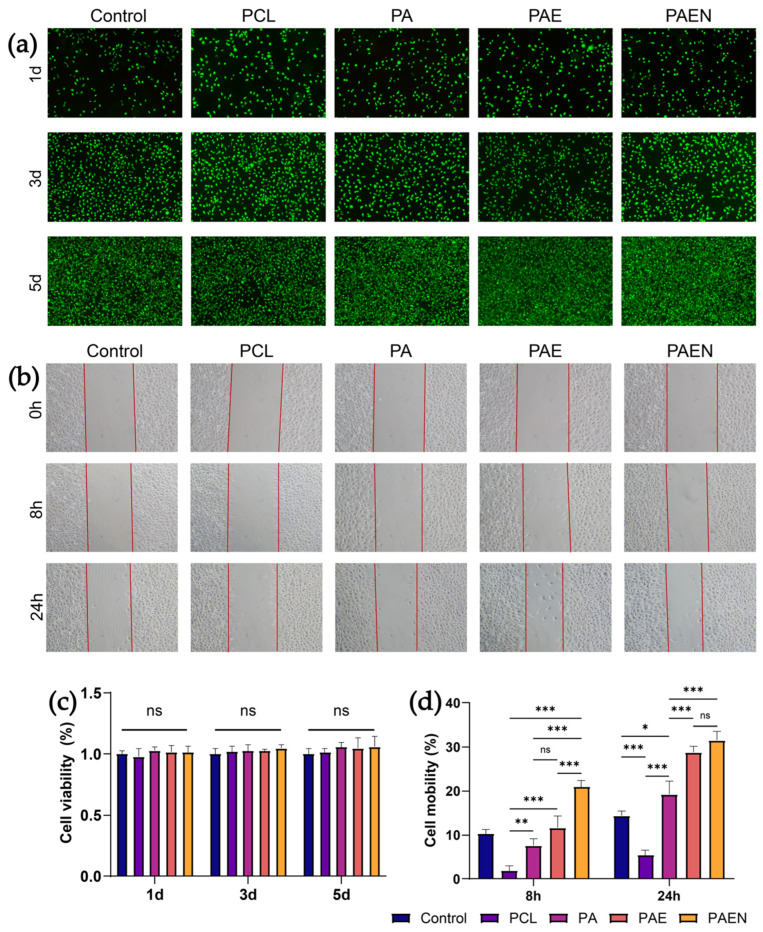
In vitro biocompatibility and pro-migratory effects of the nanofiber scaffolds. (**a**) Live/Dead staining images of fibroblasts cultured on different scaffolds for 1, 3, and 5 days. Green fluorescence indicates live cells. (**b**) Representative images of the wound healing scratch assay showing fibroblast migration at 0, 8, and 24 h. (**c**) Quantitative analysis of cell viability using the CCK-8 assay at 1, 3, and 5 days. (**d**) Quantitative analysis of cell mobility from the scratch assay at 8 and 24 h. (Data are presented as mean ± SD (*n* = 3). * *p* < 0.033, ** *p* < 0.002, *** *p* < 0.001. ns, not significant.) Note: PCL: pure polycaprolactone; PA: PCL/Az-CS; PAE: PCL/Az-CS with 7 wt% dECM; PAEN: the final PCL/Az-CS/dECM/NAC multifunctional scaffold.

The biocompatibility and effects of the scaffolds on cellular behavior were evaluated via CCK-8 and scratch migration assays (Figure 5a,b). All groups exhibited excellent cytocompatibility over 5 days (Figure 5c), with no statistically significant differences in cell viability compared to the blank control (1.000 ± 0.046). Notably, the PCL/AZ-CS/dECM/NAC scaffold maintained high relative viability (1.057 ± 0.085), confirming that neither the materials nor their leachables are cytotoxic.

In contrast, scratch assays revealed significant disparities in fibroblast migration (Figure 5d). After 24 h, the hydrophobic PCL scaffold inhibited migration (5.449 ± 1.167%) compared to the control (14.457 ± 1.012%). However, the introduction of bioactive components incrementally enhanced pro-migratory effects: migration rates increased to 19.320 ± 2.935% (PCL/AZ-CS) and 28.824 ± 1.328% (PCL/AZ-CS/dECM), with the PCL/AZ-CS/dECM/NAC scaffold demonstrating the most potent activity (31.540 ± 2.100%). These trends highlight the modulation of cell behavior by the scaffold’s surface microenvironment. While the hydrophobicity of PCL hinders cell adhesion and spreading—thus impeding migration—the integration of AZCS improves surface wettability and biocompatibility. The further acceleration in the PCL/AZ-CS/dECM and PCL/AZ-CS/dECM/NAC groups is attributed to dECM, which provides a protein-rich, biomimetic substrate and a more hydrophilic surface that collectively drive cellular motility.

### 3.4. In Vitro Anti-Inflammatory and Antioxidant Capacity of the Nanofiber Scaffolds

**Figure 6:**
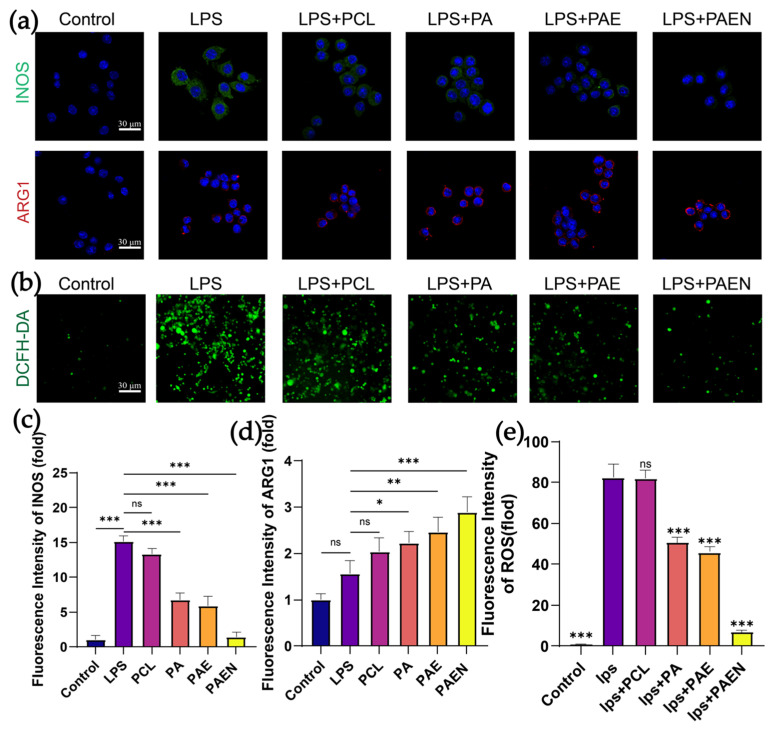
Scaffolds promote M2 macrophage polarization and scavenge intracellular ROS. (**a**) Immunofluorescence staining for M1 (iNOS) and M2 (ARG1) markers in LPS-stimulated macrophages. (**b**) Detection of intracellular ROS levels. (**c**–**e**) Corresponding quantitative analysis of the fluorescence intensity for (**c**) iNOS, (**d**) ARG1, and (**e**) ROS. (Data are presented as mean ± SD (*n* = 3). * *p* < 0.033, ** *p* < 0.002, *** *p* < 0.001. ns, not significant.) Note: PCL: pure polycaprolactone; PA: PCL/Az-CS; PAE: PCL/Az-CS with 7 wt% dECM; PAEN: the final PCL/Az-CS/dECM/NAC multifunctional scaffold.

M1 macrophages produce large amounts of reactive oxygen species and nitric oxide, which exacerbates the inflammatory effect [66]. In diabetic wounds, the persistent M1 polarization of macrophages is a key reason for impaired healing. In contrast, M2 macrophages are involved in suppressing inflammation and promoting tissue repair and angiogenesis [67]. To deeply investigate the immunomodulatory mechanism by which the composite scaffolds promote wound healing, we evaluated their effect on macrophage polarization via immunofluorescence staining, focusing on the expression of the M1 (pro-inflammatory) phenotype marker iNOS and the M2 (pro-reparative) phenotype marker ARG1 [68] (Figure 6a). Both the LPS-stimulated group (iNOS: 15.13 ± 0.801) and the pure PCL scaffold group (iNOS: 13.34 ± 0.800) exhibited a strong pro-inflammatory response (Figure 6c). However, the introduction of AZCS marked a crucial turning point in the immunomodulatory behavior of the materials. Compared to the PCL group, the PCL/AZ-CS scaffold significantly suppressed iNOS expression down to 6.703 ± 1.037 while beginning to promote ARG1 expression (2.227 ± 0.238) (Figure 6d). This trend continued in the PCL/AZ-CS/dECM group, where iNOS expression dropped further to 5.833 ± 1.396 and ARG1 rose to 2.457 ± 0.315; however, these changes were not statistically significant compared to the PCL/AZ-CS group. The PCL/AZ-CS/dECM/NAC composite scaffold showed the strongest immunomodulatory capacity, bringing iNOS levels down nearly to baseline (1.360 ± 0.750) while pushing ARG1 expression to its peak (2.900 ± 0.327).

The intracellular ROS scavenging capacity of the scaffolds was evaluated using the DCFH-DA probe (Figure 6b,e). Unstimulated macrophages showed minimal baseline fluorescence (1.000 ± 0.095), whereas LPS stimulation induced a drastic elevation in ROS levels (82.43 ± 6.545). PCL had little impact on oxidative stress (82.03 ± 3.934, *p* > 0.12), but fluorescence dropped to 50.70 ± 2.687 and 45.98 ± 2.473 in the PCL/AZ-CS and PCL/AZ-CS/dECM groups. The strongest response was observed with the PCL/AZ-CS/dECM/NAC scaffold; it cut ROS levels by over 90% (6.880 ± 1.065), effectively bringing them back to baseline.

These results explain the mechanism behind the PCL/AZ-CS/dECM/NAC scaffold’s immunomodulatory effects. Since high intracellular ROS levels keep pro-inflammatory M1 signaling active, neutralizing ROS is essential for shifting macrophage polarization. By clearing the ROS triggered by LPS, the PCL/AZ-CS/dECM/NAC scaffold removes a major signal for the M1 phenotype, effectively breaking the inflammatory cycle. This process explains the link between the suppression of iNOS and the strong upregulation of ARG1 observed in our study. Consequently, the PCL/AZ-CS/dECM/NAC scaffold serves as an active microenvironmental regulator, eliminating oxidative stress to drive macrophage polarization from a pro-inflammatory M1 to a pro-reparative M2 phenotype.

### 3.5. Wound Healing Ability of Nanofiber Scaffolds

**Figure 7:**
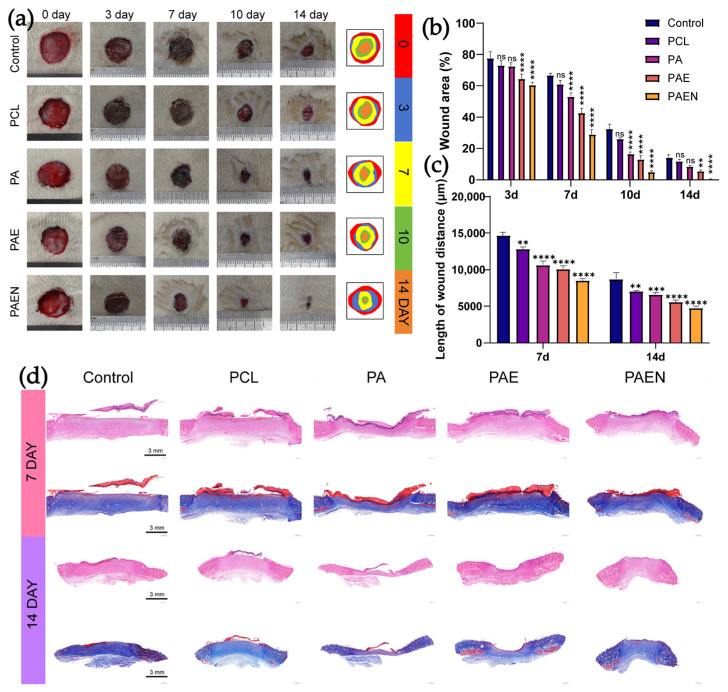
In vivo wound healing efficacy of the nanofiber scaffolds in a diabetic rat model. (**a**) Representative macroscopic images of the wounds on days 0, 3, 7, 10, and 14. (**b**) Quantitative analysis of the wound closure rate over time. (**c**) Measurement of the distance between the epithelial margins on days 7 and 14. (**d**) H&E and Masson’s trichrome staining of the regenerated tissues on days 7 and 14. (Scale bar = 3 mm). (Data are presented as mean ± SD (*n* = 3). ** *p* < 0.002, *** *p* < 0.0002, **** *p* < 0.0001, ns, not significant.) Note: PCL: pure polycaprolactone; PA: PCL/Az-CS; PAE: PCL/Az-CS with 7 wt% dECM; PAEN: the final PCL/Az-CS/dECM/NAC multifunctional scaffold.

To assess therapeutic efficacy, a diabetic full-thickness wound model was monitored over 14 days (Figure 7a,b). Preliminary observations indicated that healing was significantly impaired under diabetic conditions. By day 14, the control and PCL groups retained 14.350 ± 1.326% and 11.927 ± 1.038% of their wound areas, respectively. In contrast, dECM incorporation appeared to accelerate healing. Notably, at day 7, the PCL/AZ-CS/dECM/NAC group showed a trend toward superior performance with a remaining area of 29.060 ± 2.921%, compared to 42.737 ± 3.119% for the PCL/AZ-CS/dECM group. This accelerating effect was further observed in later stages; the PCL/AZ-CS/dECM/NAC group reached nearly complete closure by day 10 (5.020 ± 0.825%) and achieved almost total re-epithelialization by day 14 (0.543 ± 0.160%), outperforming all other treatments.

We measured the gap between wound edges on H&E-stained sections to preliminarily quantify re-epithelialization across the different groups (Figure 7c,d). By day 7, distinct trends in the healing rate had emerged. The control group showed the slowest regeneration, with a wound distance of 14,631.50 ± 458.45 μm. Adding components to the scaffold led to a steady improvement in healing; the distance narrowed to 12,806.57 ± 278.48 μm for PCL and reached 10,094.30 ± 429.78 μm for the PCL/AZ-CS/dECM group. The PCL/AZ-CS/dECM/NAC group stood out, achieving the fastest re-epithelialization with a gap of only 8475.97 ± 285.73 μm. This gap between the PCL/AZ-CS/dECM/NAC and other groups widened further by day 14. While the control wounds still measured 8745.43 ± 859.53 μm, the PCL/AZ-CS/dECM/NAC-treated wounds had closed significantly to 4747.33 ± 254.58 μm, far exceeding the performance of any other group.

Our in vivo results generally align with what we observed in vitro. The slow healing in the control and PCL groups illustrates the reality of the diabetic wound environment, where high inflammation and oxidative stress likely prevent cells from dividing or moving. By adding AZCS and dECM, we not only made the scaffold more hydrophilic but also introduced biological signals that help recruit cells. Specifically, the PCL/AZ-CS/dECM/NAC scaffold was observed to clear ROS to lower the oxidative burden, creating a much safer space for cell survival. The enhanced efficacy of the PCL/AZ-CS/dECM/NAC scaffold is likely attributed to its components complementing each other. It does more than just provide a physical frame; it transforms the harsh wound bed into a pro-healing environment, allowing for faster skin regrowth. These findings highlight PCL/AZ-CS/dECM/NAC as a practical solution for the complex problems of diabetic wound care.

### 3.6. In Vivo Anti-Inflammatory Capacity of Nanofiber Scaffolds

**Figure 8:**
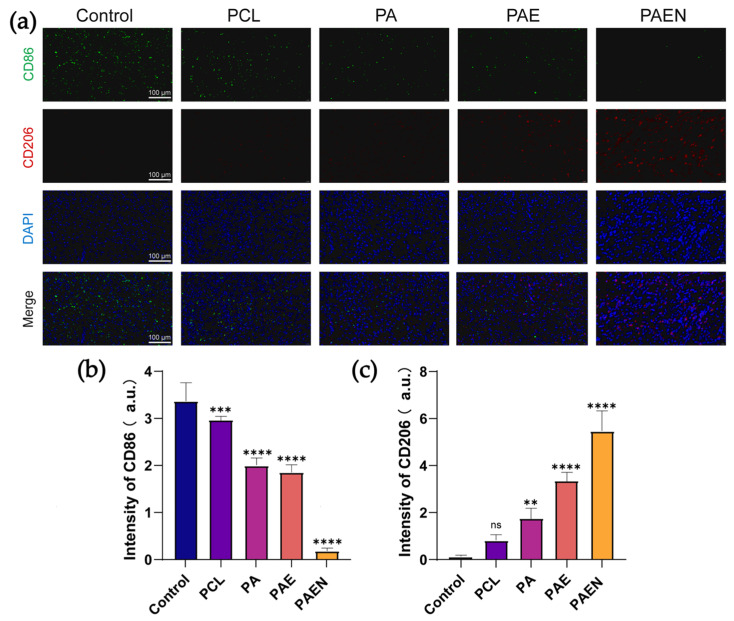
The PCL/AZ-CS/DECM/NAC scaffold promotes an M1-to-M2 macrophage phenotype transition in vivo. (**a**) Representative immunofluorescence staining of wound tissues on day 14 for M1 and M2 macrophage markers. (**b**,**c**) Quantitative analysis of the fluorescence intensity of (**b**) CD86 and (**c**) CD206 expression. (Data are presented as mean ± SD (*n* = 3). ** *p* < 0.002, *** *p* < 0.0002, **** *p* < 0.0001, ns, not significant.) Note: PCL: pure polycaprolactone; PA: PCL/Az-CS; PAE: PCL/Az-CS with 7 wt% dECM; PAEN: the final PCL/Az-CS/dECM/NAC multifunctional scaffold.

To preliminarily validate the immunomodulatory effect of the composite scaffolds on the wound microenvironment in vivo, we performed immunofluorescence staining for M1 (CD86) and M2 (CD206) macrophage phenotypes on tissue sections from day 14 (Figure 8a). Observations showed that both the control and PCL scaffold groups exhibited a notable presence of the pro-inflammatory M1 phenotype (CD86 expression of 3.363 ± 0.392 and 2.130 ± 0.282 a.u., respectively) with minimal expression of the M2 marker CD206 (Figure 8b,c). This trend suggests that in the absence of effective intervention, the persistent oxidative stress in the wound likely contributes to a sustained pro-inflammatory macrophage phenotype. In contrast, the PCL/AZ-CS and PCL/AZ-CS/dECM scaffolds appeared to reduce M1 polarization (CD86 reduced to 0.997 ± 0.158 and 0.953 ± 0.160 a.u., respectively) while promoting M2 polarization. The PCL/AZ-CS/dECM/NAC scaffold group showed the strongest immunomodulatory trend, likely due to its superior antioxidant capacity. In this group, the M1 signal was observed to be substantially lower (CD86: 0.110 ± 0.060 a.u.), while the M2 signal appeared to reach its highest level (CD206: approx. 7.0 a.u.). These results provide evidence suggesting how the PCL/AZ-CS/dECM/NAC scaffold may help mitigate the inflammatory loop by effectively clearing excess ROS from the wound site. This shift indicates a potential transition of macrophages from a pro-inflammatory M1 state to a pro-healing M2 phenotype. This immune-regulating effect offers a possible explanation for the accelerated healing observed with the PCL/AZ-CS/dECM/NAC scaffold.

### 3.7. Histological Evaluation and Analysis of Scar Formation in Wound Regeneration

**Figure 9:**
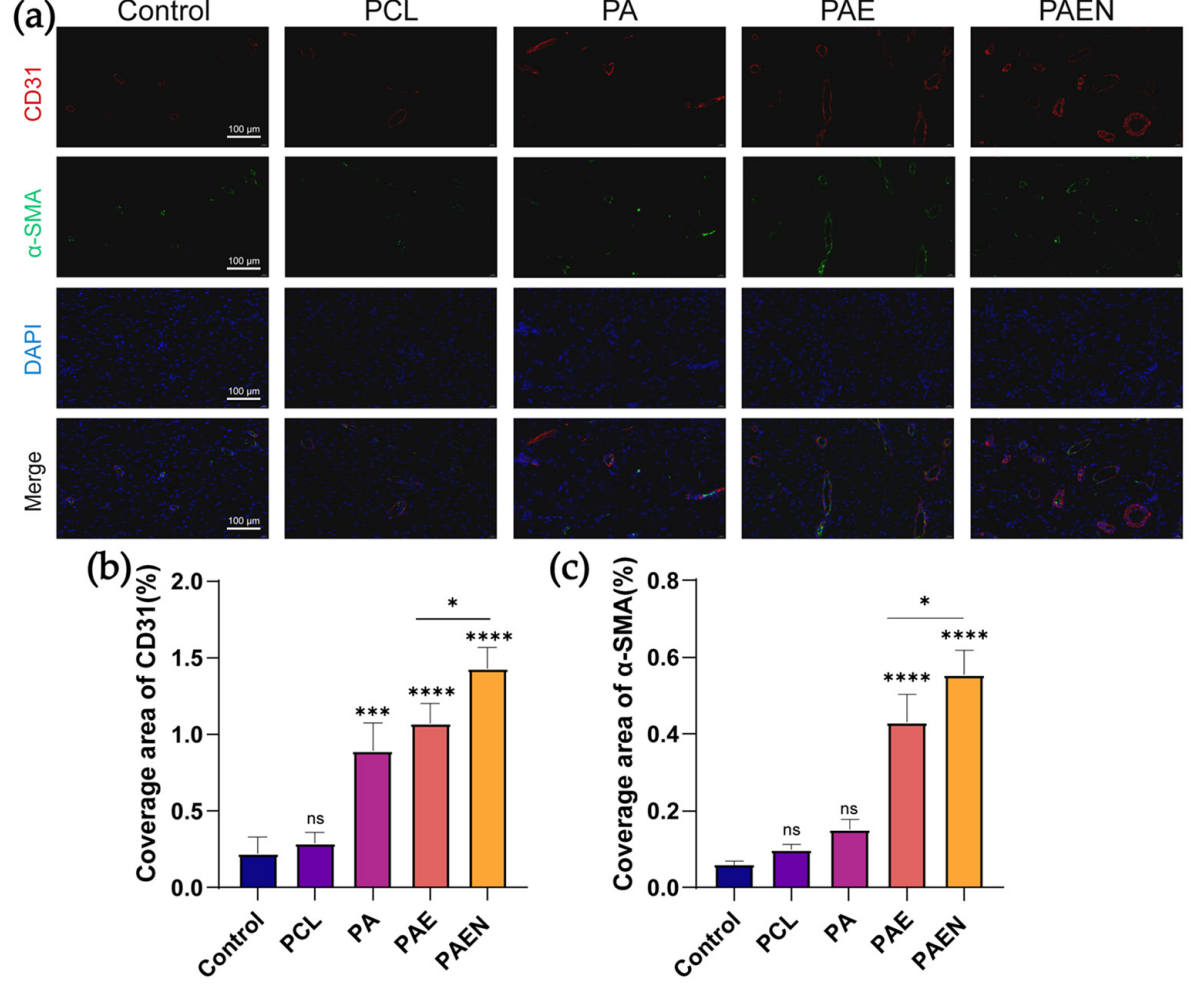
Immunofluorescence analysis of neovascularization and vascular maturation within the regenerated wound tissues on day 14. (**a**) Representative confocal microscopy images showing the expression and co-localization of the endothelial cell marker CD31 (red) and the pericyte/smooth muscle cell marker α-SMA (green). (**b**,**c**) Semi-quantitative analysis of the percentage coverage area of (**b**) CD31 and (**c**) α-SMA. (Data are presented as mean ± SD (*n* = 3). * *p* < 0.033, *** *p* < 0.0002, **** *p* < 0.0001, ns, not significant.) Note: PCL: pure polycaprolactone; PA: PCL/Az-CS; PAE: PCL/Az-CS with 7 wt% dECM; PAEN: the final PCL/Az-CS/dECM/NAC multifunctional scaffold.

To preliminarily investigate the underlying cellular mechanisms driving this regeneration, immunofluorescence staining was performed for key markers of angiogenesis (CD31) and vascular maturation (α-SMA) (Figure 9a). Angiogenesis is crucial for delivering oxygen and nutrients to the regenerating tissue. Quantitative analysis of CD31 (Figure 9b) indicated that the coverage area of neovasculature in the PCL/AZ-CS/dECM/NAC-treated group (1.40 ± 0.11%) appeared higher than that in all other groups, including the PCL/AZ-CS/dECM (1.08 ± 0.09%) and PCL/AZ-CS (0.89 ± 0.10%) groups, while the control and PCL groups remained at extremely low levels. These observations suggest the pro-angiogenic potential of the PCL/AZ-CS/dECM/NAC scaffold. Concurrently, we evaluated the expression of α-SMA (Figure 9c), which is closely associated with vascular maturation and tissue remodeling. The observed trend for α-SMA generally aligned with that of CD31: it increased from the extremely low levels in the control and PCL groups to 0.42 ± 0.08% in the PCL/AZ-CS/dECM group, and reached a peak of 0.55 ± 0.06% in the PCL/AZ-CS/dECM/NAC group. Observation of the immunofluorescence co-localization images revealed that α-SMA-positive cells (green) were primarily found enveloping or adjacent to the CD31-positive neovasculature (red). These preliminary findings suggest that the increase in α-SMA may reflect the recruitment of pericytes and smooth muscle cells to the neovasculature, a key hallmark of vascular network maturation and stabilization [69].

**Figure 10:**
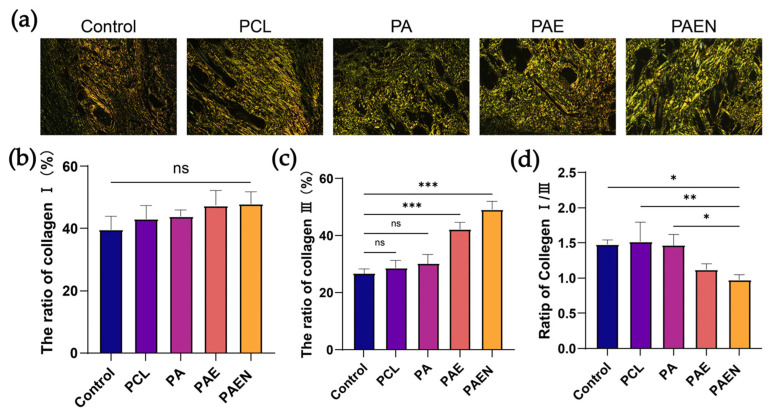
Collagen deposition and remodeling in the regenerated wound tissues on day 14. (**a**) Representative images of Picrosirius Red-stained tissue sections under polarized light, showing the morphology and organization of collagen fibers (Type I collagen: orange/red; Type III collagen: green/yellow). (**b**–**d**) Corresponding quantitative analysis of (**b**) the ratio of Type I collagen, (**c**) the ratio of Type III collagen, and (**d**) the Collagen I/III ratio. (Data are presented as mean ± SD (*n* = 3). * *p* < 0.033, ** *p* < 0.002, *** *p* < 0.0002. ns, not significant).

We evaluated the impact of the scaffolds on collagen remodeling and scar quality using Picrosirius Red staining (Figure 10a). Under polarized light, the control and PCL groups showed sparse, disorganized collagen fibers. In contrast, the PCL/AZ-CS/dECM/NAC-treated group appeared to form a dense, well-ordered network, with fiber bundles arranged in a healthy basket-weave pattern resembling native dermis. While Type I collagen levels remained similar across all groups (Figure 10b), a noticeable trend was observed in Type III collagen, which reached approximately 48% in the PCL/AZ-CS/dECM/NAC group (Figure 10c). This rebalancing appeared to lower the Collagen I/III ratio—a key metric for scar assessment [70]. Specifically, this ratio dropped below 1.0 in the PCL/AZ-CS/dECM/NAC group, representing a notable difference compared to the ~1.5 ratio observed in the control and PCL groups (Figure 10d).

Overall, these histological results suggest that the PCL/AZ-CS/dECM/NAC composite scaffold may actively guide tissue regeneration. Instead of just speeding up wound closure, the scaffold uses immunomodulation to support both vessel growth and the deposition of a Type III collagen-rich matrix. This process could contribute to high-quality skin reconstruction with minimal scarring.

## 4. Conclusions

This study developed a multi-component nanofiber scaffold to accelerate wound recovery and minimize scar formation. In vivo results indicated that the PCL/AZ-CS/dECM/NAC scaffold drives cell migration, angiogenesis, and high-quality tissue repair through its specific biological activities. By shifting macrophage polarization toward the pro-reparative M2 phenotype, the scaffold was observed to help suppress the chronic inflammation that typically leads to fibrosis, thereby potentially limiting scar development at its source. Its antioxidant and hydrophilic properties appeared to stabilize the wound microenvironment, protecting cells from oxidative stress and prolonged inflammation. Histological data suggested that the PCL/AZ-CS/dECM/NAC scaffold organizes collagen remodeling by boosting the proportion of Type III collagen and optimizing the Collagen I/III ratio. Together, in vitro and in vivo findings highlight the potential that this multifunctional scaffold offers as a viable approach to solving the dual challenge of slow healing and excessive scarring in regenerative medicine.

Despite the promising results, this study has certain limitations. The in vivo animal experiments were conducted with a relatively small sample size (*n* = 3 per group). While the observed differences between groups were statistically significant and consistent across multiple time points, these findings should be regarded as preliminary. Future studies with larger animal cohorts are necessary to fully validate the long-term clinical potential and statistical robustness of the PCL/Az-CS/dECM/NAC scaffold.

## Data Availability

The original contributions presented in this study are included in the article and Supplementary Materials. Further inquiries can be directed to the corresponding authors.

## Supplementary Materials

The following supporting information can be downloaded at https://www.mdpi.com/article/10.3390/polym18040525/s1: Figure S1: Mechanical characterization of the scaffolds; Figure S2: Standard curve of NAC content in PBS.

## References

[1] Mony M.P., Harmon K.A., Hess R., Dorafshar A.H., Shafikhani S.H. (2023). An Updated Review of Hypertrophic Scarring. Cells.

[2] Ekstein S.F., Wyles S.P., Moran S.L., Meves A. (2020). Keloids: A review of therapeutic management. Int. J. Dermatol..

[3] Reinholz M., Poetschke J., Schwaiger H., Epple A., Ruzicka T., Gauglitz G.G. (2015). The dermatology life quality index as a means to assess life quality in patients with different scar types. J. Eur. Acad. Dermatol. Venereol..

[4] Hawash A.A., Ingrasci G., Nouri K., Yosipovitch G. (2021). Pruritus in Keloid Scars: Mechanisms and Treatments. Acta Derm.-Venereol..

[5] Kassi K., Kouame K., Kouassi A., Allou A., Kouassi I., Kourouma S., Ecra E., Sangare A. (2020). Quality of life in black African patients with keloid scars. Dermatol. Rep..

[6] Kim S.W. (2022). Management of keloid scars: Noninvasive and invasive treatments. Arch. Plast. Surg..

[7] Dasari N., Jiang A., Skochdopole A., Chung J., Reece E.M., Vorstenbosch J., Winocour S. (2021). Updates in Diabetic Wound Healing, Inflammation, and Scarring. Semin. Plast. Surg..

[8] Gabriel V. (2011). Hypertrophic Scar. Phys. Med. Rehabil. Clin..

[9] Wallace H.A., Basehore B.M., Zito P.M. (2017). Wound Healing Phases.

[10] Almadani Y.H., Vorstenbosch J., Davison P.G., Murphy A.M. (2021). Wound Healing: A Comprehensive Review. Semin. Plast. Surg..

[11] Volpe C.M.O., Villar-Delfino P.H., dos Anjos P.M.F., Nogueira-Machado J.A. (2018). Cellular death, reactive oxygen species (ROS) and diabetic complications. Cell Death Dis..

[12] Fatehi-Hassanabad Z., Chan C.B., Furman B.L. (2010). Reactive oxygen species and endothelial function in diabetes. Eur. J. Pharmacol..

[13] Rendra E., Riabov V., Mossel D.M., Sevastyanova T., Harmsen M.C., Kzhyshkowska J. (2019). Reactive oxygen species (ROS) in macrophage activation and function in diabetes. Immunobiology.

[14] Dunnill C., Patton T., Brennan J., Barrett J., Dryden M., Cooke J., Leaper D., Georgopoulos N.T. (2015). Reactive oxygen species (ROS) and wound healing: The functional role of ROS and emerging ROS-modulating technologies for augmentation of the healing process. Int. Wound J..

[15] He X., Young S.-H., Schwegler-Berry D., Chisholm W.P., Fernback J.E., Ma Q. (2011). Multiwalled Carbon Nanotubes Induce a Fibrogenic Response by Stimulating Reactive Oxygen Species Production, Activating NF-κB Signaling, and Promoting Fibroblast-to-Myofibroblast Transformation. Chem. Res. Toxicol..

[16] Alili L., Sack M., Puschmann K., Brenneisen P. (2014). Fibroblast-to-myofibroblast switch is mediated by NAD(P)H oxidase generated reactive oxygen species. Biosci. Rep..

[17] Deng L., Du C., Song P., Chen T., Rui S., Armstrong D.G., Deng W., Ciobica A. (2021). The Role of Oxidative Stress and Antioxidants in Diabetic Wound Healing. Oxidative Med. Cell. Longev..

[18] Fan X., Huang J., Zhang W., Su Z., Li J., Wu Z., Zhang P. (2024). A Multifunctional, Tough, Stretchable, and Transparent Curcumin Hydrogel with Potent Antimicrobial, Antioxidative, Anti-inflammatory, and Angiogenesis Capabilities for Diabetic Wound Healing. ACS Appl. Mater. Interfaces.

[19] Zhu W., Dong Y., Xu P., Pan Q., Jia K., Jin P., Zhou M., Xu Y., Guo R., Cheng B. (2022). A composite hydrogel containing resveratrol-laden nanoparticles and platelet-derived extracellular vesicles promotes wound healing in diabetic mice. Acta Biomater..

[20] Zhang P., He L., Zhang J., Mei X., Zhang Y., Tian H., Chen Z. (2020). Preparation of novel berberine nano-colloids for improving wound healing of diabetic rats by acting Sirt1/NF-κB pathway. Colloids Surf. B Biointerfaces.

[21] Xiang T., Guo Q., Jia L., Yin T., Huang W., Zhang X., Zhou S. (2023). Multifunctional Hydrogels for the Healing of Diabetic Wounds. Adv. Healthc. Mater..

[22] Akbik D., Ghadiri M., Chrzanowski W., Rohanizadeh R. (2014). Curcumin as a wound healing agent. Life Sci..

[23] Yin J., Qin S., Chen J., Wong N., Peng C., Li D. (2025). Berberine-based strategies: Novel delivery systems bring out new potential for wound healing. Chin. Med..

[24] Zgheib C., Hilton S.A., Dewberry L.C., Hodges M.M., Ghatak S., Xu J., Singh S., Roy S., Sen C.K., Seal S. (2019). Use of Cerium Oxide Nanoparticles Conjugated with MicroRNA-146a to Correct the Diabetic Wound Healing Impairment. J. Am. Coll. Surg..

[25] Shanker K., Naradala J., Mohan G.K., Kumar G.S., Pravallika P.L. (2017). A sub-acute oral toxicity analysis and comparative in vivo anti-diabetic activity of zinc oxide, cerium oxide, silver nanoparticles, and Momordica charantia in streptozotocin-induced diabetic Wistar rats. RSC Adv..

[26] Piyush More S.G., Soham Jagtap R.N., Chippalkatti R. (2015). Antidiabetic and Antioxidant Properties of Copper Nanoparticles Synthesized by Medicinal Plant Dioscorea bulbifera. J. Nanomed. Nanotechnol..

[27] Javed B., Ikram M., Farooq F., Sultana T., Mashwani Z.-u.-R., Raja N.I. (2021). Biogenesis of silver nanoparticles to treat cancer, diabetes, and microbial infections: A mechanistic overview. Appl. Microbiol. Biotechnol..

[28] Xu L., Wang Y.-Y., Huang J., Chen C.-Y., Wang Z.-X., Xie H. (2020). Silver nanoparticles: Synthesis, medical applications and biosafety. Theranostics.

[29] Woźniak-Budych M.J., Staszak K., Staszak M. (2023). Copper and Copper-Based Nanoparticles in Medicine—Perspectives and Challenges. Molecules.

[30] Dong Y., Wang Z. (2023). ROS-scavenging materials for skin wound healing: Advancements and applications. Front. Bioeng. Biotechnol..

[31] Junker J.P.E., Kamel R.A., Caterson E.J., Eriksson E. (2013). Clinical Impact Upon Wound Healing and Inflammation in Moist, Wet, and Dry Environments. Adv. Wound Care.

[32] Maver T., Hribernik S., Mohan T., Smrke D.M., Maver U., Stana-Kleinschek K. (2015). Functional wound dressing materials with highly tunable drug release properties. RSC Adv..

[33] Jun I., Han H.-S., Edwards J., Jeon H. (2018). Electrospun Fibrous Scaffolds for Tissue Engineering: Viewpoints on Architecture and Fabrication. Int. J. Mol. Sci..

[34] Zhu X., Cui W., Li X., Jin Y. (2008). Electrospun fibrous mats with high porosity as potential scaffolds for skin tissue engineering. Biomacromolecules.

[35] Wu J., Hong Y. (2016). Enhancing cell infiltration of electrospun fibrous scaffolds in tissue regeneration. Bioact. Mater..

[36] Mulholland E.J. (2020). Electrospun Biomaterials in the Treatment and Prevention of Scars in Skin Wound Healing. Front. Bioeng. Biotechnol..

[37] Chen Z., Xiao L., Hu C., Shen Z., Zhou E., Zhang S., Wang Y. (2023). Aligned lovastatin-loaded electrospun nanofibers regulate collagen organization and reduce scar formation. Acta Biomater..

[38] Zhang H., Guo M., Zhu T., Xiong H., Zhu L.-M. (2022). A careob-like nanofibers with a sustained drug release profile for promoting skin wound repair and inhibiting hypertrophic scar. Compos. Part B Eng..

[39] Jiang Z., Zheng Z., Yu S., Gao Y., Ma J., Huang L., Yang L. (2023). Nanofiber Scaffolds as Drug Delivery Systems Promoting Wound Healing. Pharmaceutics.

[40] Mochane M.J., Motsoeneng T.S., Sadiku E.R., Mokhena T.C., Sefadi J.S. (2019). Morphology and Properties of Electrospun PCL and Its Composites for Medical Applications: A Mini Review. Appl. Sci..

[41] Lv C., Dai H., Xing X., Zhang J. (2012). The systematic effects of chitosan on fibroblasts derived from hypertrophic scars and keloids. Indian J. Dermatol. Venereol. Leprol..

[42] Kim S. (2018). Competitive Biological Activities of Chitosan and Its Derivatives: Antimicrobial, Antioxidant, Anticancer, and Anti-Inflammatory Activities. Int. J. Polym. Sci..

[43] Cady E., Orkwis J.A., Weaver R., Conlin L., Madigan N.N., Harris G.M. (2020). Micropatterning Decellularized ECM as a Bioactive Surface to Guide Cell Alignment, Proliferation, and Migration. Bioengineering.

[44] Aktunc E., Ozacmak V.H., Ozacmak H.S., Barut F., Buyukates M., Kandemir O., Demircan N. (2010). N-acetyl cysteine promotes angiogenesis and clearance of free oxygen radicals, thus improving wound healing in an alloxan-induced diabetic mouse model of incisional wound. Clin. Exp. Dermatol..

[45] Liu S., Zhao Y., Li M., Nie L., Wei Q., Okoro O.V., Jafari H., Wang S., Deng J., Chen J. (2023). Bioactive wound dressing based on decellularized tendon and GelMA with incorporation of PDA-loaded asiaticoside nanoparticles for scarless wound healing. Chem. Eng. J..

[46] Hur W., Park M., Lee J.Y., Kim M.H., Lee S.H., Park C.G., Kim S.-N., Min H.S., Min H.J., Chai J.H. (2016). Bioabsorbable bone plates enabled with local, sustained delivery of alendronate for bone regeneration. J. Control. Release.

[47] Yang H., Liu Y., Wen F., Yan X., Zhang Y., Zhong Z. (2024). Preparation, characterization, antioxidant and antifungal activities of benzoic acid compounds grafted onto chitosan. Int. J. Biol. Macromol..

[48] Allman A.J., McPherson T.B., Badylak S.F., Merrill L.C., Kallakury B., Sheehan C., Raeder R.H., Metzger D.W. (2001). Xenogeneic extracellular matrix grafts elicit a TH2-restricted immune response1. Transplantation.

[49] Zhang Y., Tan J., Chen Y. (2023). Visible-light-induced protein labeling in live cells with aryl azides. Chem. Commun..

[50] Bumgardner J.D., Wiser R., Elder S.H., Jouett R., Yang Y., Ong J.L. (2003). Contact angle, protein adsorption and osteoblast precursor cell attachment to chitosan coatings bonded to titanium. J. Biomater. Sci. Polym. Ed..

[51] Geng X., Kwon O.-H., Jang J. (2005). Electrospinning of chitosan dissolved in concentrated acetic acid solution. Biomaterials.

[52] Honarpardaz A., Irani S., Pezeshki-Modaress M., Zandi M., Sadeghi A. (2018). Enhanced chondrogenic differentiation of bone marrow mesenchymal stem cells on gelatin/glycosaminoglycan electrospun nanofibers with different amount of glycosaminoglycan. J. Biomed. Mater. Res. Part A.

[53] Yue B. (2014). Biology of the Extracellular Matrix. J. Glaucoma.

[54] Chen Z.G., Wang P.W., Wei B., Mo X.M., Cui F.Z. (2010). Electrospun collagen–chitosan nanofiber: A biomimetic extracellular matrix for endothelial cell and smooth muscle cell. Acta Biomater..

[55] Kalra A., Lowe A., Al-Jumaily A.M. (2016). Mechanical Behaviour of Skin: A Review. J. Mater. Sci. Eng..

[56] Menzies K.L., Jones L. (2010). The impact of contact angle on the biocompatibility of biomaterials. Optom. Vis. Sci..

[57] Lin N., Zuo B. (2021). Silk sericin/fibroin electrospinning dressings: A method for preparing a dressing material with high moisture vapor transmission rate. J. Biomater. Sci. Polym. Ed..

[58] Jonidi Shariatzadeh F., Currie S., Logsetty S., Spiwak R., Liu S. (2025). Enhancing wound healing and minimizing scarring: A comprehensive review of nanofiber technology in wound dressings. Prog. Mater. Sci..

[59] Akash M.S.H., Rehman K. (2020). Drug Stability and Chemical Kinetics.

[60] Dima C., Pătraşcu L., Cantaragiu A., Alexe P., Dima Ş. (2016). The kinetics of the swelling process and the release mechanisms of Coriandrum sativum L. essential oil from chitosan/alginate/inulin microcapsules. Food Chem..

[61] Ukaegbu K., Allen E., Svoboda K.K.H. (2025). Reactive Oxygen Species and Antioxidants in Wound Healing: Mechanisms and Therapeutic Potential. Int. Wound J..

[62] Sindrilaru A., Peters T., Wieschalka S., Baican C., Baican A., Peter H., Hainzl A., Schatz S., Qi Y., Schlecht A. (2011). An unrestrained proinflammatory M1 macrophage population induced by iron impairs wound healing in humans and mice. J. Clin. Investig..

[63] Dong F., Xue Q., Liu J., Guo Z., Zhong H., Sun H. The influence of amino and hydroxyl of chitosan on hydroxyl radical scavenging activity. Proceedings of the 2009 3rd International Conference on Bioinformatics and Biomedical Engineering.

[64] Pei Y., Liu H., Yang Y., Yang Y., Jiao Y., Tay F.R., Chen J., Czuczejko J. (2018). Biological Activities and Potential Oral Applications of N-Acetylcysteine: Progress and Prospects. Oxidative Med. Cell. Longev..

[65] Tenório M.C.d.S., Graciliano N.G., Moura F.A., Oliveira A.C.M.d., Goulart M.O.F. (2021). N-Acetylcysteine (NAC): Impacts on Human Health. Antioxidants.

[66] Tan H.-Y., Wang N., Li S., Hong M., Wang X., Feng Y., Venditti P. (2016). The Reactive Oxygen Species in Macrophage Polarization: Reflecting Its Dual Role in Progression and Treatment of Human Diseases. Oxidative Med. Cell. Longev..

[67] Jetten N., Verbruggen S., Gijbels M.J., Post M.J., De Winther M.P.J., Donners M.M.P.C. (2013). Anti-inflammatory M2, but not pro-inflammatory M1 macrophages promote angiogenesis in vivo. Angiogenesis.

[68] Lisi L., Ciotti G.M.P., Braun D., Kalinin S., Currò D., Dello Russo C., Coli A., Mangiola A., Anile C., Feinstein D.L. (2017). Expression of iNOS, CD163 and ARG-1 taken as M1 and M2 markers of microglial polarization in human glioblastoma and the surrounding normal parenchyma. Neurosci. Lett..

[69] Zohar B., Blinder Y., Mooney D.J., Levenberg S. (2017). Flow-Induced Vascular Network Formation and Maturation in Three-Dimensional Engineered Tissue. ACS Biomater. Sci. Eng..

[70] Wang C., Rong Y., Ning F., Zhang G. (2011). The content and ratio of type I and III collagen in skin differ with age and injury. Afr. J. Biotechnol..

