# Supplementary material for: A Biomimetic NAC-Loaded PCL/Modified Chitosan/dECM Fibrous Scaffold for Accelerating Diabetic Wound Healing and Minimizing Scarring
Source: Polymers (Basel). 2026 Feb 20;18(4):525. doi: 10.3390/polym18040525 (PMC12944638; doi:10.3390/polym18040525)
Supplement: Supplementary file 1 [file polymers-18-00525-s001.zip › polymers-4148764-supplementary.pdf]

Supporting Information

A Biomimetic NAC-Loaded PCL/Modified Chitosan/dECM Fibrous Scaffold for Accelerating Diabetic Wound Healing and Minimizing Scarring

Yiju Xie [1,2], Banchao Ruan [3], Yihua Yin [1,4], Lihong Fan [1,4,*], Haolin Tang [1,2], Heshuang Dai [1,4,*], Sasha You [1,2], Shiyuan Yao [1,4], Guangxu Wang [1,4] and Yihan Xu [1,4]

[1] Sanya Science and Education Innovation Park of Wuhan University of Technology, Sanya 572024, China; 348315@whut.edu.cn (Y.X.); yihuayin@aliyun.com (Y.Y.); thln@whut.edu.cn (H.T.); 358846@whut.edu.cn (S.Y.); 348337@whut.edu.cn (S.Y.); 348334@whut.edu.cn (G.W.); 348336@whut.edu.cn (Y.X.)
[2] School of Materials Science and Engineering, Wuhan University of Technology, Wuhan 430070, China
[3] BSc Chemistry, Department of Chemistry,University of Liverpool, Liverpool L69 3BX, UK; sgbruan@liverpool.ac.uk
[4] School of Chemistry, Chemical Engineering and Life Sciences, Wuhan University of Technology, Wuhan 430070, China
* Correspondence: lhfan@whut.edu.cn (L.F.); daiheshuang@whut.edu.cn (H.D.)

1. Methods

1.1. Physical characterization of electrospun fibrous scaffolds

1.1.1. Scanning electron microscopy (SEM) and fiber diameter distribution

The morphology of the fibrous scaffolds was examined using scanning electron microscopy (SEM). SEM images were captured at 50,000× magnification. For each sample, the average fiber diameter was determined by measuring 100 randomly selected fibers from the SEM images using ImageJ software.

Academic Editor: Young-Sam Cho

Received: 27 January 2026
Revised: 17 February 2026
Accepted: 19 February 2026
Published: 20 February 2026

Copyright: © 2026 by the authors. Licensee MDPI, Basel, Switzerland. This article is an open access article distributed under the terms and conditions of the Creative Commons Attribution (CC BY) license.

1.1.2. Mechanical property testing

The electrospun fibrous scaffolds were cut into small strips (30×10 mm^2). Uniaxial tensile tests were performed using a mechanical testing machine. The gauge length of the sample between the clamps was approximately 10 mm. During the tensile test, the specimens were stretched to failure at an elongation rate of 10 mm/min. The tensile strength, Young's modulus, and elongation at break of the scaffolds were obtained from the resulting stress-strain curves. Each sample type was measured in triplicate, and the average results were calculated.

1.1.3. Water contact angle measurement of electrospun fibrous scaffolds

Hydrophilicity was assessed using a contact angle goniometer (FCA2000A3E, Aifeisi Precision Instruments Co., Ltd., Shanghai). A 2 μL droplet of deionized water was deposited onto the surface of the material, and an image was immediately captured by the instrument. To ensure accuracy and consistency, each sample was measured in triplicate, and the average result was calculated.

1.1.4. Water vapor transmission rate of electrospun fibrous scaffolds

To measure the water vapor transmission rate, the nanofibrous scaffolds were sealed over the opening of glass vials (1.73×27.5cm) containing 10 mL of phosphate-buffered saline (PBS). The vials were placed in an incubator at 37 °C and weighed every 24 hours to determine the amount of water loss. The WVTR was

calculated according to the following formula:

$$WVTR\ (g/m^2/day) = \Delta W/A/24$$

where ΔW is the change in mass (g), A is the sample area (m^2), and T is the time interval (days).

1.1.5. Water absorption capacity of electrospun fibrous scaffolds

The initial dry weight of the samples was recorded as W_0. The samples were then immersed in PBS at room temperature. At predetermined time intervals, the samples were removed, excess surface water was blotted off, and the wet weight (W_t) was measured. The water absorption ratio (SR) at time t was calculated using the following formula:

$$SR = \frac{W_t - W_0}{W_t} \times 100\%$$

1.1.6. Porosity of electrospun fibrous scaffolds

The porosity of the nanofibers was evaluated using the ethanol displacement method. A dry sample was weighed, then immersed in absolute ethanol for 2 hours. Subsequently, the sample was removed, and the wet sample was weighed. All measurements were performed in triplicate. The porosity was calculated using the following formula:

$$Porosity\ (\%) = \frac{(W_{wet} - W_{dry}) \times 100\ \%}{(\rho_{\mathrm{ethanol}} \times V_{\mathrm{scaffold}})}$$

where W_{wet} is the weight of the wet sample, W_{dry} is the weight of the dry sample, $\rho_{ethanol}$ is the density of ethanol, and $V_{scaffold}$ is the volume of the dry scaffold.

1.2. *In vitro* cell studies

1.2.1. Cytotoxicity assay of the electrospun fibrous scaffolds

Cytocompatibility of the prepared fiber scaffolds was evaluated with L929 mouse fibroblasts using the CCK-8 assay. The scaffolds were cut into circular discs (8 mm in diameter), sterilized by immersion in 75% (v/v) ethanol, and subsequently rinsed with sterile PBS. L929 cells were seeded onto the scaffolds at a density of 1×10^4 cells/mL in 48-well plates (Corning) and cultured for 1, 3, and 5 days. At each predetermined time point, 10 μL of CCK-8 reagent was added to each well, and the plates were incubated for an additional 4 hours in a CO_2 incubator. To further assess cell viability, a Calcein-AM/PI double staining kit (Shanghai Zeye Biological Technology Co., Ltd.) was employed. Live cells were stained green with Calcein-AM, while dead cells exhibited red fluorescence due to propidium iodide (PI). The stained cells were then visualized using a confocal laser scanning microscope (CLSM, Leica TCS SP5 II, Germany).

1.2.2. Fibroblast migration assay

L929 cells were seeded into 96-well plates and co-cultured with the fiber scaffolds at 37°C for 24 h. Following the incubation, a linear scratch was created on the confluent cell monolayer using a 10 μL pipette tip. The wells were rinsed twice with PBS to remove detached cells and debris, after which 100 μL of fresh culture medium was added. The initial scratch width (0 h) was documented using an optical microscope. The plates were then returned to the incubator, and the scratch area was recorded at designated time intervals (0, 8, and 24 h). The migration distance was quantified using ImageJ software, and the migration rate was calculated according to the following equation:

$$Migration\, rate\ (\%) = \frac{A_0 - A_t}{A_0} \times 100\%$$

where A_0 represents the scratch area at 0 h, and A_t represents the scratch area at 8 h or 24 h.

1.2.3 *In vitro* evaluation of anti-inflammatory and antioxidant properties of fibrous scaffolds

RAW264.7 cells were stimulated with 1 μg/mL lipopolysaccharide (LPS) for 12 h, followed by co-culture with PCL, PA, PAE, and PAEN nanofibrous scaffolds. Untreated cells served as the control group. After 3 days of culture, the cells were harvested and stained for two key polarization markers: inducible nitric oxide synthase (iNOS, for M1 phenotype) and arginase-1 (Arg1, for M2 phenotype). The fluorescence signals were observed and captured using a confocal laser scanning

microscope (CLSM, Leica TCS SP5 II, Germany).

The antioxidant capacity of the four nanofibrous scaffolds was evaluated using a DCFH2-DA probe. RAW264.7 cells were seeded in 6-well plates at a density of 1.0×10^6 cells/well and incubated overnight for attachment. The cells were then pre-treated with 1 μg/mL LPS for 4 h, followed by a 1 h incubation with various scaffold extracts at 37°C. Untreated cells were used as the negative control. Subsequently, the cells were incubated with 10 μM DCFH2-DA in a 37°C incubator for 30 min. The fluorescence intensity, reflecting the level of intracellular reactive oxygen species (ROS), was recorded at an excitation wavelength of 480 nm using an inverted fluorescence microscope (Nikon, Japan).

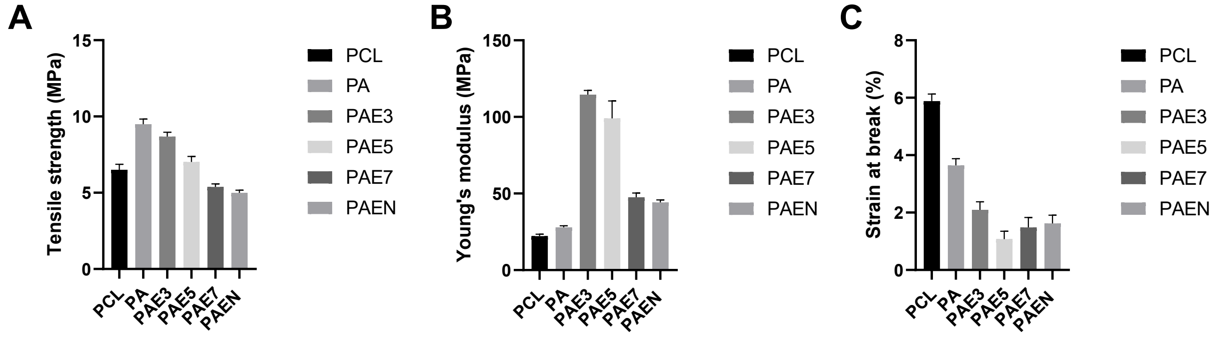

Figure S1. Mechanical characterization of the scaffolds. (A) Tensile strength, (B) Young's modulus, and (C) strain at break of PCL, PA, PAE series, and PAEN nanofibers. Data are presented as mean ± standard deviation (n=3).

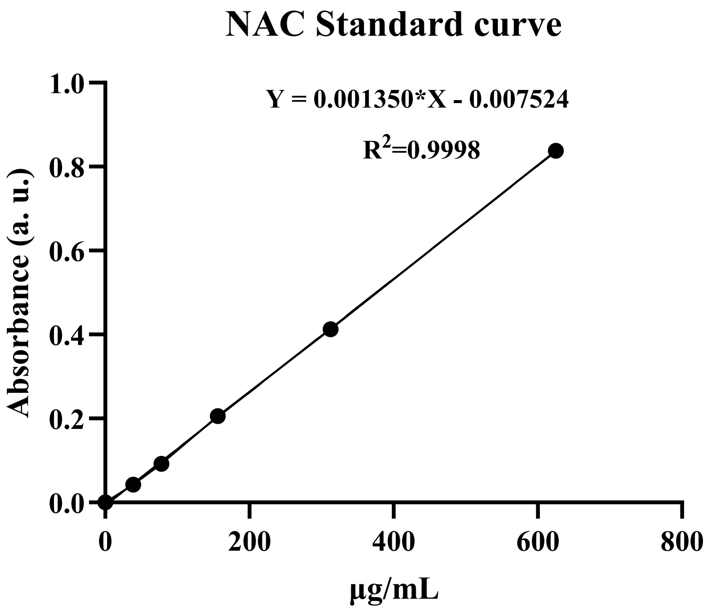

Figure S2. Standard curve of NAC content in PBS
